# Gastric Glomus Tumor: A Rare Cause of Acute Blood Loss Anemia

**DOI:** 10.7759/cureus.24511

**Published:** 2022-04-26

**Authors:** Faisal Mehmood, Hajra Jamil, Amina Khalid

**Affiliations:** 1 Hospital Medicine, Montefiore Medical Center, New York City, USA; 2 Internal Medicine, Services Institute of Medical Sciences, Lahore, PAK; 3 Hospital Medicine, Albert Einstein College of Medicine, New York City, USA

**Keywords:** smooth-muscle actin, gastrointestinal stromal tumor (gist), coffee-ground emesis, gastric glomus tumors, acute blood loss anemia

## Abstract

Gastric glomus tumors (GGTs) are rare mesenchymal neoplasms that arise from cells of the glomus body. These occur in the submucosa of the gastric wall and are usually benign in nature. However, it is difficult to predict tumor behavior due to the lack of reliable histological features. Diagnosis can be challenging due to the lack of specific clinical features, and radiologic and endoscopic findings. Computed tomography (CT) scan, esophagogastroduodenoscopy (EGD), and endoscopic ultrasound (EUS) are key diagnostic modalities. However, the final diagnosis depends on the postoperative immunohistochemical and pathological analysis. Most GGTs can be cured by surgical or endoscopic resection. We report a case of GGT in a middle-aged woman who presented with new-onset anemia and was found to have a gastric mass that was later diagnosed as GGT after immunohistochemical staining.

## Introduction

Gastrointestinal stromal tumors (GISTs) are the most common mesenchymal neoplasms of the gastrointestinal tract. These typically affect older individuals and most commonly involve the stomach [1]. Gastric glomus tumors (GGTs) account for approximately 1% of all GISTs [2]. These are far more common in women in the sixth and seventh decades of life than in men [3,4]. The gastric antrum is the most commonly involved site. Clinical presentation is non-specific and can present with epigastric discomfort, nausea, vomiting, hematemesis, or melena [3,4]. In rare cases, it can be an incidental diagnosis [4]. Computed tomography (CT) scan, esophagogastroduodenoscopy (EGD), and endoscopic ultrasound (EUS) are key diagnostic modalities. Most patients are diagnosed postoperatively after immunohistochemical and pathological analysis. We report a case of GGT in a middle-aged woman who presented with dizziness and pre-syncope episodes in the setting of new-onset anemia and was found to have a gastric mass on EGD, requiring laparoscopic partial gastrectomy and later diagnosed as GGT after immunohistochemical staining.

This case was presented as a meeting abstract at the American College of Gastroenterology Annual Scientific Meeting in Las Vegas on October 26, 2021.

## Case presentation

A 50-year-old woman presented to the hospital with dizziness and pre-syncope episodes for the past several days. She also reported one episode of coffee ground emesis and black stools. She did not report dysphagia, odynophagia, heartburn, abdominal pain, early satiety, weight loss, diarrhea, constipation, or hematochezia. She denied taking non-steroidal anti-inflammatory drugs (NSAIDs), iron pills, or Pepto-Bismol (Procter & Gamble Company, Cincinnati, Ohio, United States). She never had an EGD before. She was a non-smoker. Her medical history was remarkable for obesity, hypertension, and coronary artery disease, and she was taking aspirin at home.

**Figure 1:**
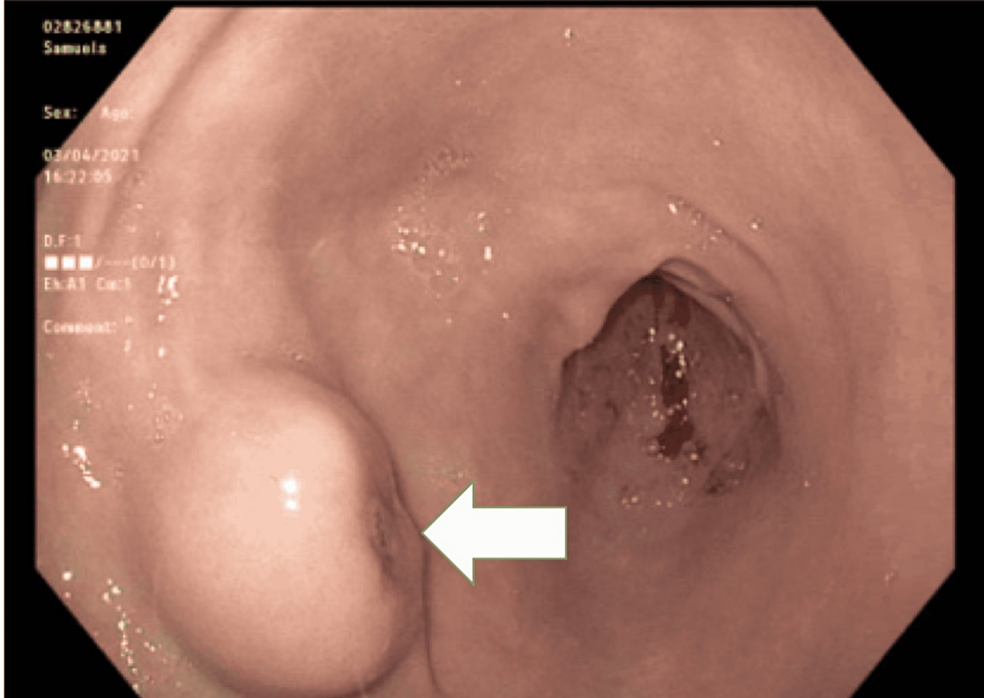
Esophagogastroduodenoscopy showing a 3cm round mass with small central area of ulceration (white arrow) in gastric antrum EGD: esophagogastroduodenoscopy

On examination, she was hemodynamically stable and without abdominal tenderness. A rectal exam revealed brown stools and the guaiac test was positive. Laboratory reports were remarkable for normocytic anemia with a hemoglobin of 4.9 g/dL from a baseline of 10 g /dL with a normal platelet count. Other blood tests including liver test, coagulation profile, and kidney function were normal. She was given intravenous pantoprazole and three packed red blood cells (pRBCs) units with an appropriate response with repeat hemoglobin of 8.4 g/dL. CT abdomen and pelvis with IV contrast showed no abdominal mass and scattered colonic diverticula without evidence of acute diverticulitis. However, evaluation of the stomach was limited by its under distension. She underwent an EGD which showed a 3cm round mass with a small central area of ulceration in the gastric antrum concerning for gastric malignancy (Figure 1). Gastric mass biopsy was inconclusive showing chronic inflammation with lymphoid aggregate, negative for *Helicobacter pylori* and any malignant cells. She underwent a CT chest for staging purposes and it was negative for metastasis.

**Figure 2:**
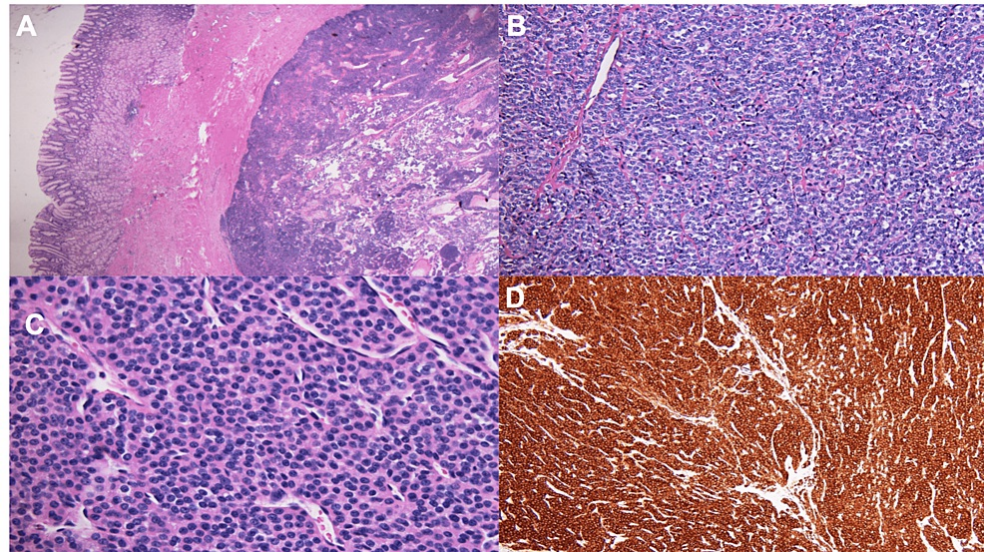
H&E staining of GGT: (A) 2x magnification showing a well-circumscribed cellular neoplasm in muscularis propria; (B) 20x magnification; and (C) 40x magnification showing nests of small, uniform, and rounded cells surrounding capillary-sized vessels. No cytologic atypia seen. (D) Immunohistochemical study showing the tumor cells strongly positive for SMA H&E: Hematoxylin and eosin; GGT: gastric glomus tumor; SMA: smooth muscle actin

The case was discussed on the tumor board, and it was decided to pursue a diagnostic laparoscopy with a repeat upper endoscopy. There was no evidence of liver or peritoneal metastasis on laparoscopy. Laparoscopic partial gastrectomy was performed. The specimen was sent to pathology for frozen evaluation and the preliminary result was consistent with an epithelioid type tumor concerning for a GIST or neuroendocrine tumor. Tumor margins were negative. Final surgical pathology revealed a tumor size of 1.6 cm in the greatest dimension. Tumor cells were strongly positive for smooth muscle actin (SMA), pericellular collagen type IV, and desmin. The tumor cells were negative for AE1/AE3, S100, inhibin, melan-A, and chromogranin. Synaptophysin, CD117, and STAT6 show non-specific background staining. She was diagnosed with a GGT based on immunohistochemical analysis (Figure 2). There was no indication of any adjuvant therapy. 

**Figure 3:**
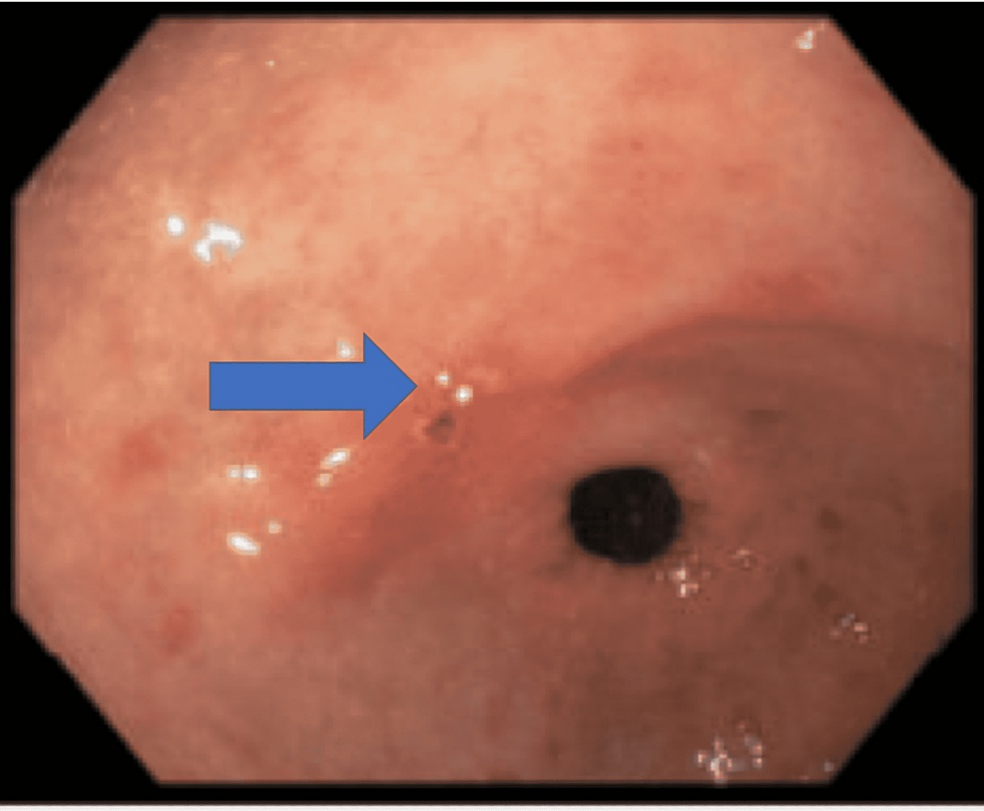
EGD showing a 4mm localized erosion (Blue arrow) with overlying eschar in the gastric antrum in the previous site of known glomus tumor EGD: esophagogastroduodenoscopy

She did well postoperatively and was discharged home in a stable condition. Eight months later, she had a repeat CT scan of the abdomen and pelvis with no evidence of visceral metastasis. EGD was performed one year after the resection and showed a 4mm localized erosion with overlying eschar in the gastric antrum in the previous site of the known glomus tumor (Figure 3). A biopsy was sent and did not reveal any significant histopathologic abnormality. *Helicobacter pylori* organisms were not seen on the hematoxylin and eosin (H&E) stain. She was doing well on follow-up visits.

## Discussion

The incidence of GGTs is very low as compared to GISTs with only one in 100 diagnoses of GISTs being a GGT. These are far more common in women than in men [2,3]. There is a discrepancy in information when the first case of GGT was diagnosed. Some papers report that the first case was diagnosed in 1928 while in others it is said to be 1948 and 1951 [5,6]. However, the first malignant glomus tumor of the stomach was diagnosed in 1939 [7].

Clinical presentation is non-specific and can present with epigastric discomfort, nausea, vomiting, hematemesis, or melena [3,4]. CT scan, EGD, and EUS are key diagnostic modalities. In our case, a CT scan of the abdomen did not show any lesions in the stomach concerning malignancy. However, it has been shown that GGTs manifest as smooth submucosal masses with or without ulceration. There may be some areas of calcification and exhibit strong early-phase contrast enhancement [8]. However, radiological diagnosis can be challenging due to overlapping radiological features with other gastric submucosal lesions [9]. Differential diagnosis includes carcinoid tumor, hemangioma, GIST, leiomyoma, paraganglioma, lipoma, and other rare tumors. EUS can be useful to identify the layer of origin. However, images from EUS are insufficient to establish a diagnosis of GGT without a clinicopathologic analysis [10]. The endoscopic biopsy can be difficult due to the intramural location of the tumor. However, EUS-guided biopsy has been reported to successfully diagnose GGT in some cases with cytologic and immunochemical analysis of the specimen [11].

Immunohistochemistry is helpful to differentiate it from other mesenchymal neoplasms. Positive SMA and vimentin are seen in glomus cells. Other markers including chromogranin A, neuron-specific enolase and cytokeratin, CD117 antigen including S-100 protein, CD34, CD117, desmin, CD56, and synaptophysin are negative [3]. Most GGTs are benign and can be cured by surgical or endoscopic resection. They have a good overall prognosis, but a small, unpredictable potential for malignant behavior exists [4,12]. Folpe et al. [12] suggested the criteria for defining malignancy in GGT and estimating the risk of recurrence and metastasis. The parameters for the proposed criteria include the size (greater than 2 cm), depth, and the combination of high nuclear grade and mitotic activity [> 5/50 high-power fields (HPFs)]. Tumors with a size less than 5.0 cm tend to behave in a benign fashion. Our patient had a tumor size of 1.6 cm in the greatest dimension and had likely a benign tumor. Malignant tumors are aggressive and may recur locally or metastasized to other organs. Therefore, a long-term follow-up is advised. The most common sites of metastases are the brain, bones, small intestine, lung, and liver [12,13]. 

The treatment of choice for GGTs is surgical resection of the tumor. Choice of the surgical procedure depends on the location and the size of the tumor. Surgical options include laparoscopic, open subtotal gastrectomy, or wedge resection. Complete surgical resection with negative margins (“no ink on tumor”) is sufficient with no need for further resection or lymph node dissection [14]. Laparoscopy endoscopy cooperative surgery (LECS) is a less invasive and effective option if the tumor is located near the pylorus or at the gastroesophageal junction, and growing in an endophytic pattern [15]. Due to the unpredictable potential for malignant behavior, long-term follow-up is suggested. Due to the paucity of clinical data, there are no consensus guidelines for treatment and surveillance [16,17]. In our case, there were no lesions on the CT scan suggestive of a GGT and the initial biopsy report from EGD was inconclusive. A multidisciplinary team of physicians was involved and it was decided to pursue laparoscopic partial gastrectomy with tumor resection after a shared decision with the patient. Diagnosis of GGT was based on immunohistochemical analysis postoperatively. Our patient also had a repeat CT scan of the abdomen and an EGD to monitor for malignant transformation.

## Conclusions

GGTs are rare gastrointestinal lesions that are predominately benign but there is unpredictable potential for malignant behavior. Diagnosis can be challenging due to significant overlapping features with other common stromal and mesenchymal lesions. Most cases are diagnosed postoperatively based on immunohistochemical analysis. Due to the rarity of glomus tumors and limited published knowledge, physicians should determine the best course of treatment, endoscopic or surgical intervention. A multidisciplinary team of physicians including gastroenterologists, surgeons, oncologists, and pathologists should be involved in the care of these patients. It is crucial to determine the malignant potential to pursue management options ranging from surveillance to endoscopic or surgical intervention. In our case, the patient had a repeat CT scan and an EGD, which did not reveal any recurrence or metastasis.
